# Disruption of PCNA-lamins A/C interactions by prelamin A induces DNA replication fork stalling

**DOI:** 10.1080/19491034.2016.1239685

**Published:** 2016-09-27

**Authors:** Andrew M. Cobb, Thomas V. Murray, Derek T. Warren, Yiwen Liu, Catherine M. Shanahan

**Affiliations:** King's College London, The James Black Center, London, United Kingdom

**Keywords:** DNA damage, DNA replication fork stalling, nuclear lamina, PCNA, Prelamin A

## Abstract

The accumulation of prelamin A is linked to disruption of cellular homeostasis, tissue degeneration and aging. Its expression is implicated in compromised genome stability and increased levels of DNA damage, but to date there is no complete explanation for how prelamin A exerts its toxic effects. As the nuclear lamina is important for DNA replication we wanted to investigate the relationship between prelamin A expression and DNA replication fork stability. In this study we report that the expression of prelamin A in U2OS cells induced both mono-ubiquitination of proliferating cell nuclear antigen (PCNA) and subsequent induction of Pol η, two hallmarks of DNA replication fork stalling. Immunofluorescence microscopy revealed that cells expressing prelamin A presented with high levels of colocalisation between PCNA and γH2AX, indicating collapse of stalled DNA replication forks into DNA double-strand breaks. Subsequent protein-protein interaction assays showed prelamin A interacted with PCNA and that its presence mitigated interactions between PCNA and the mature nuclear lamina. Thus, we propose that the cytotoxicity of prelamin A arises in part, from it actively competing against mature lamin A to bind PCNA and that this destabilises DNA replication to induce fork stalling which in turn contributes to genomic instability.

## Introduction

The nuclear lamina consists of type V intermediate filament proteins that are concentrated on the nucleoplasmic surface of the inner nuclear membrane.^1^ Lamins A and C are both derived from the same gene (*LMNA*), however lamin A is extensively post-translationally modified to become mature lamin A.^2,3^ Defective processing can lead to the accumulation of lamin A precursors such as progerin^4^ and prelamin A^5^ that are implicated in laminopathies Hutchinson-Gilford progeria syndrome^6^ and restrictive dermopathy^5^ respectively. Despite inducing comparable effects upon cells, progerin and prelamin A differ structurally and biochemically, with progerin being a truncated form of lamin A missing 50 internal amino acids whereas prelamin A is the full transcript from the *LMNA* gene.^7^ An important similarity is that both retain a C-terminal farnesyl residue that is usually removed during post-translational processing. Although laminopathies are rare disorders, recent evidence has suggested non-mature lamins also accumulate in cells during normal aging in the general population. In particular, prelamin A has been found to accumulate in vascular smooth muscle cells prior to senescence both *in vitro* and *in vivo*^8,9^ and its expression is associated with nuclear morphology defects,^9^ heterochromatin alterations^10^ and elevated levels of DNA damage.^11-13^ Due to its cytotoxic effects, prelamin A has been proposed to be a key factor in aging of the vasculature and age-associated cardiovascular complications.^14-17^

How non-mature lamin A causes an increase in levels of DNA damage remains to be fully established. Evidence suggests they may directly impede DNA repair by delaying recruitment of repair factors to DNA lesions.^11,18,19^ Additionally, the rate of formation of DNA damage itself may be increased which would exacerbate the toxicity of these proteins. In this respect, it has been reported that accumulation of prelamin A or the depletion of lamins A/C increases levels of reactive oxygen species that can go on to cause oxidative damage to DNA.^20^ The loss of heterochromatin, an event associated with expression of prelamin A, may also be harmful as previously compacted DNA will be more exposed to damaging agents.^21,22^

Problems with lamin-regulated DNA metabolism may also lead to the introduction of endogenously formed lesions. Indeed, lamins are recognized as being fundamental to DNA replication. Not only are they present at early S-phase replication sites^23^ but disruption of lamina organization using dominant negative mutants induces elongation arrest^24^ and depletion of lamins A/C prevents restarting of stalled replication forks.^25^ Furthermore, knockdown of lamin A impedes DNA replication in mice and this can be rescued via restoration of lamin A expression.^23^ Evidence also suggests the nuclear lamina controls the spatial and temporal arrangement of DNA replication as initiation occurs in a limited number of perinucleolar lamins A/C associated foci.^26,27^ Importantly, nuclear lamins have been shown to interact directly with replication machinery components such as DNA polymerases^28^ and the sliding-clamp proliferating cell nuclear antigen (PCNA).^29-31^ PCNA forms a homotrimeric-ring structure that provides a sliding clamp for DNA polymerase δ and is important for maintaining its association between the polymerase and DNA.^32^ Protein interactions with PCNA are highly complex but are principally governed by post-translational modifications, accessory binding-factors and differential binding properties of interacting partners. The interactions that occur between lamins and PCNA mediate positioning of PCNA on chromatin^30^ and mutations within *LMNA* can compromise this interaction and dramatically reduce binding affinity *in vitro*.^30^

Replication forks that encounter lesions in DNA or non-B DNA structures are liable to stalling.^33^ Ultraviolet (UV) light generates bulky photoproducts in DNA such as pyrimidine dimers that efficiently halt advancement of the replication fork.^34,35^ Stalling may also occur if stable protein-DNA complexes are encountered or if interactions that govern the integrity and progression of the DNA replication complex are compromised.^36^ Cytotoxic double-stand breaks (DSBs) can arise in the DNA if replication cannot be recovered and the stalled fork collapses. A hallmark of arrested DNA replication is the mono-ubiquitination at K164 of PCNA by Rad18,^37,38^ that is required for the recruitment of specialized DNA polymerases such as Polη that displace Pol δ or Pol ε.^39,40^ Stalled replication forks can be identified by colocalisation of PCNA and Pol η, with these sites being markedly increased following UV-irradiation.^37^ Importantly, this modification is not restricted to DNA replication that has encountered DNA lesions but can occur at all sites of stalled replication.^41^

We wanted to assess whether the accumulation of prelamin A affected the stability of DNA replication, and if instability could give rise to increased levels of DNA damage by replication fork collapse. Herein we show that prelamin A induces stalling of DNA replication forks and leads to increased levels of PCNA at sites of DNA damage, indicating collapse of DNA replication forks. We went on to find no relationship between prelamin A and levels of UV-specific DNA damage that may have caused DNA replication to fail, however we did find that prelamin A could interact with PCNA and that interactions between PCNA and lamins A/C were decreased in the presence of prelamin A despite no reductions in overall DNA synthesis. These findings provide evidence that prelamin A induces stalling of DNA replication forks that could eventually collapse and produce toxic DNA lesions.

## Materials and methods

### Cell culture / DNA damage treatments

Osteosarcoma cells (U2OS) were obtained from American Tissue Culture Collection. Cells were passaged after reaching 70% confluency and maintained in DMEM complete media (Sigma) supplemented with 10 units/mL penicillin, 10 mg/mL streptomycin, 200 mM L-glutamine and 10% FBS (or 0.5% FBS during serum starvation experiments). For DSB DNA damage induction, cells were typically treated for 3 h with 1 µM doxorubicin or etoposide. UV-irradiation was performed using a UV Stratalinker^®^ 1800 (Stratagene^®^) typically at 40 J/m^2^ unless stated. For localized UV damage, 0.5 µm membrane filters (Isopore™) were used. For BrdU incorporation, 10 µM BrdU was added to media for 2 h at 37°C.

### Adenoviral constructs and transfections

U2OS cells at 70% confluence were infected with FLAG-tagged recombinant adenoviruses containing either an uncleavable form of prelamin A mutated within the Zmpste24 cleavage site (L647R) (UCLA), or EGFP control (EGFP). Multiplicity of infection was 5 particles per cell, routinely achieving >80% transduction efficiency as assessed by control EGFP.

### Small interfering RNA–mediated interference

Dharmacon smart pool Face1 or control small interfering RNA (siRNA) oligonucleotides were transfected into U2OS cells with the use of HiPerfect transfection reagent (Qiagen). Transfected cells were left for 72 h prior to experiments.

### Antibodies and immunofluorescence

Primary antibodies used were as follows: prelamin A (SC-6214, C-20), lamin A/C (SC-6215, N-18), FLAG (M2, F3165) (Sigma); γ-H2AX (2577), PCNA (PC10) (Cell Signaling Technology, Danvers, Mass); Emerin (Novocastra); Kap1 (ab70369), γ-Tubulin (ab11316), Pol η (ab17725) and (ab180703), USP3 (ab82935), Thymine dimer (ab10347), BrdU (ab6326), H2AX (ab11175) (abcam); Ki-67 (VP-K452) (Vector Laboratories).

For immunofluorescence, cells were cultured on coverslips and were fixed in 4% paraformaldehyde in PBS followed by 3 min permeabilisation with 0.5% NP-40 in PBS or 100% methanol at −20°C for 10 min. For BrdU analysis, cells were incubated in 2M HCl for 40 min at room temperature (RT) prior to permeabilisation. Coverslips were then incubated with blocking solution (3% BSA in PBS) for 1 h at RT. Primary antibodies in blocking solution were then applied for 12 h at 4°C, followed by 1 h RT incubation with fluorescent dye conjugated secondary antibodies (Invitrogen). Coverslips were washed with PBS, mounted onto slides with medium containing DAPI, and visualized using a Leica SP5 confocal microscope.

### Thymine dimer dot blot assay

Cells were washed with ice old PBS, then scrapped into 500 µl ice old PBS and centrifuged at 1500 rcf for 5 min. Cells were then resuspended in 400 µl ice cold PBS and sonicated for 10 s. Genomic DNA was extracted using chloroform/phenol extraction and purified and concentrated by ethanol precipitation. 1000 ng of DNA was pipetted directly onto Biodyne B Nylon membrane (Pierce) and baked at 80°C for 2 h. The membrane was then blocked with 5% milk in TBST and probed with anti-thymine dimer antibody.

### Fluorescence-activated cell sorting (FACS)

Cells were trypsinised, washed once in ice-cold PBS, then resuspended in 300 µl PBS and 700 µl 100% ethanol and kept at 4°C overnight. Cells were then centrifuged and the resulting pellet resuspended in Propidium iodide stain (50ug/ml PI in PBS containing 5 units/ml RNAse A) for at least 3 h in the dark. Cell-cycle status was measured on a Flow cytometer with a minimum of 20,000 events measured per sample analyzed. FlowJo software (FlowJo, LLC) was used for analysis.

### Two step cell cycle analysis

Cell cycle analysis was performed using a Nucleocounter NC-3000 (Chemometec). Program ‘Two-step cell cycle assay’ was used according to the manufacturers instructions.

### GST precipitation

GST Pull-down assays were performed as previously described.^42^

### Flag-tag precipitation assays

U2OS cells were transduced with EGFP or UCLA as described above. Cell lysates were obtained by sonicating cells in IP buffer (10 mM Tris pH 7.5, 150 mM NaCl, 1 mM EDTA, 1% Triton X-100, protease inhibitors) and collecting supernatant after centrifugation. ANTI-Flag® M2 Affinity Gel slurry (Sigma) was added to 400 µg of protein and IP buffer was added to a final volume of 500 µl. Samples were incubated at 4°C, rotating for 2 h. Bead-protein complexes were washed 3x in IP buffer and finally the pellet was resuspended in 4x sample buffer, heated at 100°C for 10 min and analyzed by western blot.

### Co-immunoprecipitation

Cells were scrapped into ice cold PBS and centrifuged at 1500 rcf at 4°C for 5 min. Supernatant was removed and pellets resuspended in IP buffer and sonicated for 10 s prior to centrifugation and collection of supernatant. 50 µl of Protein G beads (Sigma) were washed 3x in ice cold PBS and then added to 200 µg of cell extract for pre-clearing (50 µg of non-cleared extract was kept as ‘start lysate’). Samples were centrifuged then 1 µg of anti-PCNA, anti-Lamin A/C or non-specific antibody was added to cleared extracts which were incubated at 4°C for 12 h with gentle agitation. 100 µl of washed Protein G bead slurry was then added to extracts and were incubated at 4°C for 2 h with gentle agitation. Samples were centrifuged and washed 3x in ice cold PBS. Pellets were then resuspended in 4x sample buffer and boiled for 10 min to release captured proteins. Samples were analyzed by western blot.

### Statistical analysis

Cell and foci counts for statistical analysis were performed on >100 cells in triplicate for each control and experimental group, and results were verified in at least 3 independent experiments. Data are shown as mean and with standard errors. Statistical analysis was performed with GraphPad software, and comparisons were made with the Student unpaired *t* test.

## Results

### Prelamin A expression induces mono-ubiquitination of PCNA and recruitment of Pol η

**Figure 1.**
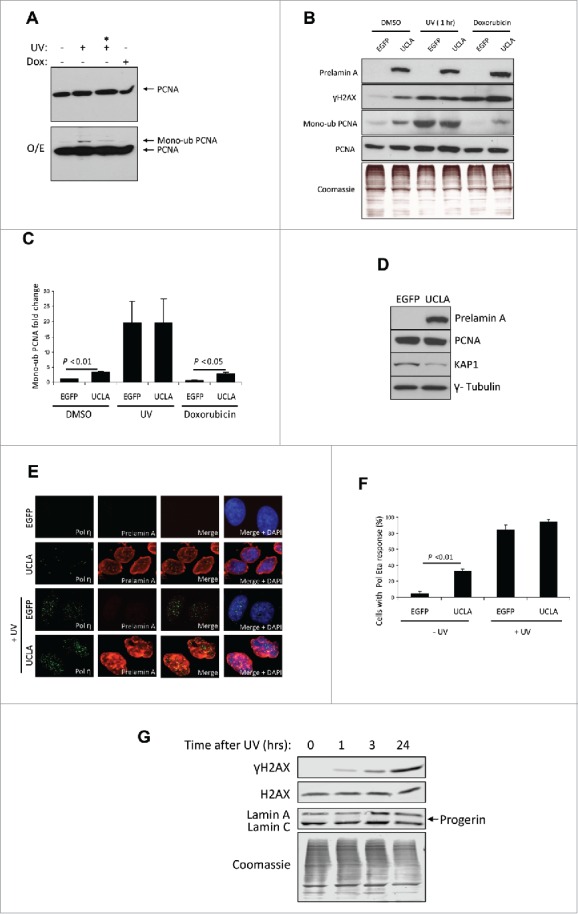
The accumulation of prelamin A (UCLA) induces mono-ubiquitination of PCNA and recruitment of polymerase η. (A) U2OS cells were subject to 40 J/m^2^ UV-irradiation and then left to recover for 1 or 3 (*) h or doxorubicin for 3 h. Nuclear fractions were then collected and analyzed by WB for analysis of nuclear PCNA. The top panel shows a blot after normal exposure and the lower panel shows a higher exposure (O/E) whereby mono-ubiquitinated species of PCNA can be seen in lysates from UV treated cells. (B) WB analysis showing U2OS cells with accumulated prelamin A contain higher endogenous levels of mono-ubiquitinated PCNA. Cells were treated with either control EGFP or UCLA (uncleavable lamin A) prior to treatment with DMSO (control), UV-irradiation or doxorubicin. Intensity of γH2AX bands reflects levels of DNA damage. The mono-ubiquitinated bands in this image were taken from the same blot as total PCNA but following a longer exposure (Supplementary information Fig. 1). Coomassie staining is shown to demonstrate equal loading. (C) Quantification of PCNA mono-ubiquitination taken from 3 experiments represented in (B) Standard errors are shown. (D) WB of U2OS whole cell lysates showing expression of prelamin A (UCLA) does not directly cause loss of PCNA but reductions in heterochromatin marker KAP1 is apparent. (E) IF showing U2OS cells expressing prelamin A (red) exhibit higher levels of Pol η (green) foci formation than EGFP controls in the absence of UV-irradiation (top 2 panel rows). Following UV-irradiation, both EGFP and UCLA expressing cells demonstrate robust Pol η foci formation (lower 2 panel rows). DAPI (blue) shows nuclear regions of cells. Cells with 3 or more Pol η foci were considered to be positive for a response. (F) Quantification of IF shown in (E) Standard errors are shown. *n* > 100 cells from 3 independent experiments. (G) WB of lysates taken from U2OS cells that had been treated with 40 J/m^2^ UV-irradiation. Lysates were collected 0, 1, 3 and 24 h post treatment. No accumulation of prelamin A or progerin was observed.

Mono-ubiquitination of K164 on PCNA by Rad18 is a primary response to replication fork stalling and can be seen following DNA bulky adduct formation. Using western blot, we were able to detect this protein modification in U2OS cells that had been treated with UV and left for 1 h (Fig. 1A). The transient nature of this response was evident as by 3 h this signal was markedly decreased. We next assessed this modification in U2OS cells that were expressing an uncleavable form of prelamin A (UCLA) against controls expressing EGFP (Fig. 1B, C and Supplementary Fig. 1). Following UV-irradiation, both cell types contained expected PCNA mono-ubiquitination, however only UCLA-expressing cells exhibited mono-ubiquitination following DMSO (vehicle control) or doxorubicin (DSB inducer) exposure, indicating prelamin A was causing DNA replication forks to stall and this was independent of UV-irradiation.

As prelamin A is associated with decreased cell proliferation, and as non-proliferating cells exhibit reduced levels of PCNA, we wondered if expression of prelamin A was having a direct influence on PCNA protein levels. We analyzed PCNA in U2OS cells expressing UCLA but saw no decrease (Fig. 1D). However, we were able to detect a decrease in KAP1, a protein associated with heterochromatin formation, supporting the surmise that prelamin A can cause a loss of heterochromatin.^43^

Following PCNA mono-ubiquitination is the recruitment of Pol η, a polymerase that is able to bypass regions of DNA that cause Pol δ to stall. We wanted to test whether prelamin A induced mono-ubiquitination of PCNA also caused this downstream event. Using immunofluorescence (Fig. 1E and F) we found that cells positive for UCLA had significantly more Pol η foci compared to controls in the absence of UV-irradiation. However, upon UV treatment, foci increased dramatically in both cell types to a similar extent.

As studies have shown UV treatment can cause expression of progerin in fibroblasts,^44^ and that progerin can inhibit repair of stalled DNA replication forks,^45^ we wanted to know if progerin could be detected in our cells before or after UV-irradiation. We were unable to detect any progerin either in non-treated U2OS cells or cells treated with UV and left from 1–24 h after irradiation prior to lysate collection (Fig. 1G), meaning the stalled DNA replication forks observed in our study were unaffected by progerin.

### γH2AX foci in cells expressing prelamin A colocalise with PCNA and Pol η

**Figure 2.**
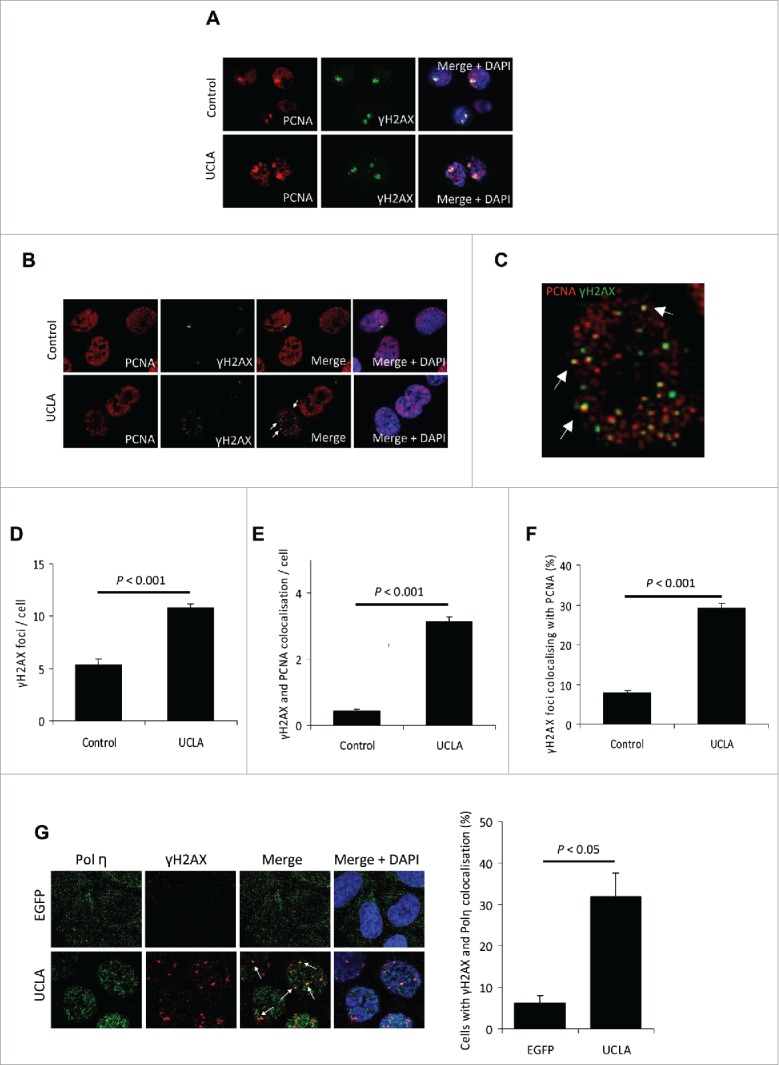
γH2AX foci present in cells expressing prelamin A colocalise with PCNA. (A) IF showing PCNA (red) and γH2AX (green) colocalise at sites of UV induced DNA damage. Expression of prelamin A (lower panel) did not affect this colocalisation. DAPI (blue) shows nuclear regions of cells. (B) IF showing γH2AX foci (green) that are present in U2OS cells expressing prelamin A (UCLA) colocalise with PCNA (red) (white arrows). (C) Increased magnification of a single U2OS cell from B, showing colocalisation of PCNA (red) and γH2AX (green). White arrows show examples of colocalisation. (D) Quantification of γH2AX foci enumeration of immunofluorescence shown in (B). *n* > 100 cells from 3 independent experiments, standard errors are shown. (E) Enumeration of γH2AX and PCNA foci colocalisation in immunofluorescence shown in (B). *n* > 100 cells from 3 experiments, standard errors are shown. (F) Quantification of percentage of γH2AX foci that colocalised with PCNA in immunofluorescence shown in (B). *n* > 100 cells from 3 independent experiments, standard errors are shown. (G) (Left) IF showing γH2AX (red) colocalises with Pol η (green) in cells expressing prelamin A (demonstrated by white arrows). DAPI is shown in blue. (Right) Quantification of γH2AX and Pol η colocalisation in cells expressing EGFP and UCLA, *n* > 100 cells from 3 independent experiments, standard errors are shown.

Cells positive for prelamin A commonly present with elevated levels of DNA damage and we hypothesized that if this DNA damage is caused by collapse of stalled DNA replication forks then there would be evidence of PCNA at DNA lesions. Initially, we ascertained by immunofluorescence microscopy that UV-irradiation caused stalling of PCNA and initiation of the DNA damage response at these sites in both control and prelamin A positive U2OS cells as evident by phosphorylation of H2AX (γH2AX) (Fig. 2A). Our next aim was to determine if regions of DNA damage observed in prelamin A positive cells independent of UV-irradiation also contained PCNA. Using immunofluorescence, we found evidence that many of the microscopically discernable foci of γH2AX present in cells expressing prelamin A colocalised with PCNA (Fig. 2B and C). Quantification of foci revealed increased γH2AX foci per cell overall (Fig. 2D), as well as a significant increase in the incidence of γH2AX and PCNA colocalisation compared to controls (Fig. 2E). Importantly, the percentage of γH2AX that colocalised with PCNA was significantly higher in cells expressing prelamin A (Fig. 2F). Analysis of cells treated with BrdU showed that cells in S-phase contained high levels of this colocalisation event (Supplementary Fig. 2), supporting that it occurs during DNA synthesis. These findings suggest that DNA damage caused by prelamin A occurs due to DNA replication stalling and eventual collapse of the fork. Moreover, we found γH2AX and PCNA colocalisation to be specific for DNA damage resulting from stalled DNA replication, as the introduction of double-strand breaks using topoisomerase inhibitors caused increases in γH2AX foci exhibiting only modest PCNA colocalisation (Supplementary Fig. S3). As further evidence that prelamin A was causing collapse of stalled DNA replication forks, we detected that the translesion synthesis polymerase Pol η was also present at sites of DNA damage (Fig. 2G).

### Prelamin A expression does not increase the incidence of thymine dimer formation

**Figure 3.**
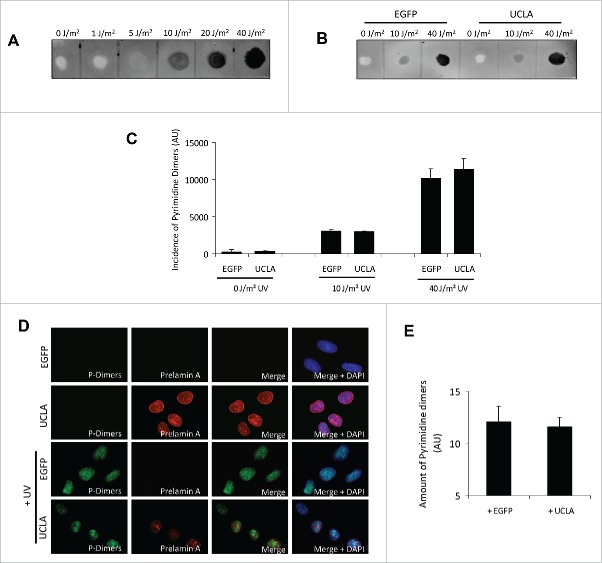
Prelamin A expression does not increase cell sensitivity to UV-irradiation. (A) Dot blot analysis of thymine dimer incidence in U2OS cells treated 0–40 J/m^2^ UV-irradiation. DNA was extracted from cells, transferred onto nylon membrane and probed with an anti-thymine dimer antibody. (B) The same technique was used to compare DNA of U2OS cells expressing EGFP or UCLA. These cells were also treated with UV to understand if UCLA influenced susceptibility to this type of DNA damage. (C) Quantification of dot intensities from assays represented in (B). Data was from 3 independent experiments, standard errors are shown. (D) IF analysis of U2OS cells expressing either EGFP (control) or UCLA (prelamin A) with or without 40 J/m^2^ UV-irradiation. Levels of thymine dimers (green) were determined in cells using an anti-thymine dimer antibody. Prelamin A (red) and DAPI (blue) are also shown. (E) Quantification of fluorescence intensity of thymine dimers in U2OS cells expressing EGFP or UCLA shown in (D). No significant differences between cells expressing EGFP or UCLA were detected. *n* > 100 cells from 3 independent experiments, standard errors are shown.

The finding that prelamin A expression leads to both mono-ubiquitination of PCNA and Pol η recruitment encouraged us to determine if stalling of DNA replication was occurring because more UV-induced DNA lesions were present. As prelamin A can cause a loss of heterochromatin,^10,46^ this could make DNA more susceptible to damage as heterochromatin has been proposed to act as a barrier to toxic stimuli such as UV-irradiation.^47,48^ To test this, we compared levels of thymine dimers in U2OS cells expressing prelamin A with control cells. Initially we utilised a dot-blot approach whereby purified genomic DNA was probed with an anti-thymine dimer antibody. As demonstrated in Fig. 3A, no thymine dimer lesions were detected in unstressed U2OS cells or cells irradiated with 1 J/m^2^ UV. However, we were able to detect lesions in cells treated with 5–40 J/m^2^. We next analyzed U2OS cells expressing EGFP or UCLA (Fig. 3B and C) that were treated with 0, 10 or 40 J/m^2^ UV. No differences at any treatments were detectable, suggesting prelamin A does not increase basal levels of thymine dimers, nor make cells more sensitive to UV light. In addition, we analyzed thymine dimers in the same cells using immunofluorescence (Fig. 3D and E) and again found that prelamin A was not causing thymine dimer formation. Taken together this data implied an alternative mechanism other than increased sensitivity to UV light was responsible for replication fork stalling in prelamin A positive cells.

### Destabilisation of mature lamina-PCNA interactions by prelamin A causes DNA damage during DNA synthesis

**Figure 4.**
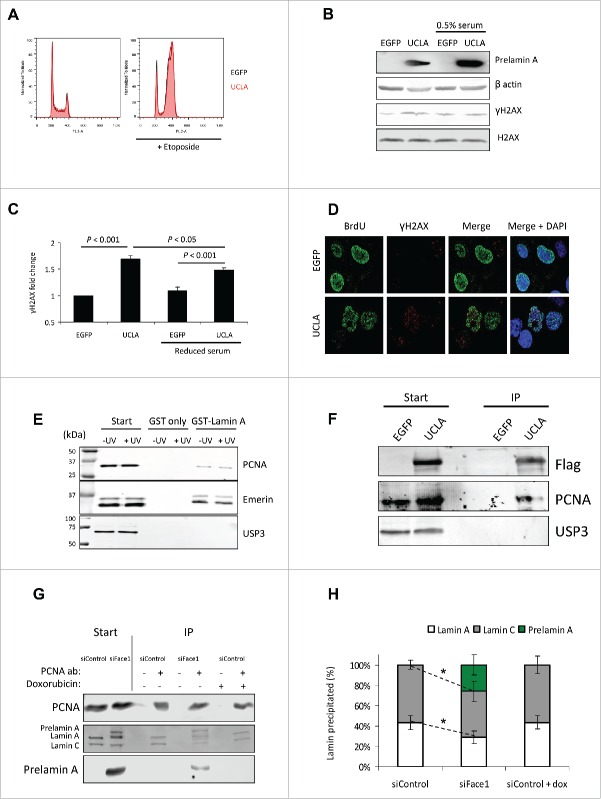
Prelamin A dependent DNA damage occurs in proliferating cells and arises concomitantly with destabilisation of PCNA interactions with mature lamin A. (A) FACS analysis of U2OS cells expressing EGFP (black) or UCLA (red). No changes in cell-cycle profiles were seen with either treatment (Supplementary Fig. 2). Treatment with etoposide (right panel) shows a significant shift to G2 arrest, but again no differences between EGFP or UCLA expression were detected. (B) WB analysis of U2OS cells expressing EGFP or UCLA that were or were not serum starved. Growing UCLA expressing U2OS cells under restricted growth conditions reduced levels of DNA damage. (C) Quantification of experiment shown in (B). Band intensities of γH2AX were taken from 4 separate experiments and normalized to EGFP control. Standard errors are shown. (D) IF showing prelamin A induces DNA damage in U2OS cells in synthesis phase. Cells were treated with EGFP or UCLA then stained for BrdU (green) and γH2AX (red). High levels of DNA damage were detected in BrdU positive cells when prelamin A was expressed (quantification shown in Fig. S5). (E) WB results of GST-pull down experiments using GST-lamin A as bait were performed to ascertain interactions with PCNA in the presence of UV-induced DNA lesions or without. GST alone was used as control. We detected Lamin A interactions with both PCNA and Emerin and both appeared to be independent of DNA damage. No interactions were seen with negative control USP3. (F) Representative WB from Flag immunoprecipitation assays investigating if PCNA interacts with prelamin A (UCLA). Following expression of EGFP (control) or UCLA in U2OS cells, recombinant proteins were immunoprecipitated using anti-Flag antibodies. (G) WB showing immunoprecipitation assays investigating if prelamin A affects PCNA interactions with lamin A/C. U2OS cells were treated with control or Face1 siRNA (prelamin A accumulation) and anti-PCNA antibody was used to precipitate PCNA and any interacting proteins. We also analyzed if DNA damage affected interactions by treating cells with doxorubicin prior to immunoprecipitation. (H) Quantification of PCNA-lamins A/C immunoprecipitation assays. Band intensities of interacting lamins were measured (Supplementary Fig. 8A) and percentages of each were calculated. It was found that prelamin A significantly reduced the amount of mature lamins A/C pulled-down with PCNA by about 25% and this was independent of DNA damage. *n* = 3, standard errors are shown, * = p < 0.05.

Photoproducts are a central inducer of PCNA mono-ubiquitination and Pol η recruitment, however other causes of DNA replication stalling can also stimulate these responses.^41^ It is recognized that the nuclear lamina is critical for DNA replication and previous studies have shown PCNA can be sequestered away from DNA replication sites by the introduction of truncated lamin proteins,^49^ however no studies have shown what impact prelamin A has upon PCNA function. We anticipated that the presence of prelamin A may adversely affect normal lamin-PCNA interactions and this may contribute to destabilisation of the DNA replication fork. As prelamin A is associated with reduced cell proliferation, we first tested whether its expression had any influence on U2OS cell growth. Fluorescence-activated cell sorting of U2OS cells expressing EGFP or UCLA revealed that after 48 h of recombinant protein expression, cell cycle profiles remained unchanged with no changes in DNA synthesis evident (Fig. 4A and Supplementary Fig. 4). We also found no differences in staining of the cell proliferation marker Ki-67 (Supplementary Fig. 5).

Our next aim was to confirm that prelamin A associated DNA damage was arising during DNA synthesis or if it was independent of proliferation status. For this, we analyzed U2OS cells expressing EGFP or UCLA that were cultured with 10% serum (proliferating) or 0.5% serum (reduced cells in S-phase (Supplementary Fig. 6)). Western blots showed a significant reduction in levels of γH2AX in prelamin A cells when proliferation was decreased under serum starved conditions (Fig. 4B and C), supporting the hypothesis that a significant proportion of DNA damage is occurring during DNA synthesis. To support this idea, we used BrdU incorporation and immunofluoresence to see if cells in S-phase contained more DNA damage. Staining of BrdU and γH2AX revealed that cells expressing prelamin A had more S-phase cells that exhibited DNA damage (Fig. 4D and Supplementary Fig. 7). In EGFP treated cells approximately 70% more non-BrdU positive cells had a γH2AX response than BrdU positive cells, however this had reversed in UCLA treated cells as more BrdU positive cells had a γH2AX response than BrdU negative cells. This data reinforces the hypothesis that prelamin A causes DNA damage in S-phase cells.

To determine if prelamin A expression affected PCNA interactions with the mature nuclear lamina we employed a GST pull-down assay to confirm PCNA interacted with mature lamin A and we successfully detected an interaction that was not affected by UV-irradiation (Fig. 4E). Following this we wanted to see if PCNA also interacted with prelamin A. For this we over-expressed EGFP and flag-tagged prelamin A in U2OS cells and precipitated these proteins using anti-flag antibodies. Analysis of precipitated proteins revealed the presence of PCNA when prelamin A was used as bait (Fig. 4F), indicating PCNA also interacts with prelamin A and thus gives rise to the possibility that prelamin A may act as a binding competitor against the mature nuclear lamina in cells with accumulated prelamin A. To further test this possibility, we used co-immunoprecipitation assays to identify if lamins A/C co-precipitation with PCNA was affected in cells treated with Face1 siRNA to accumulate prelamin A in comparison to control siRNA treated cells (Fig. 4G,H and Supplementary Fig. 8A). Depletion of Face1 significantly reduced the amount of lamins A/C precipitated by approximately 25% and this was independent of DNA damage as lamin A-PCNA interactions were not affected in cells treated with doxorubicin. The reverse IP using lamins A/C as bait also showed prelamin A was detrimental to this interaction, with less PCNA pulled-down when lamins were used as bait (Supplementary Fig. 8) These data provide evidence toward a model whereby prelamin A destabilises lamins A/C-PCNA interactions potentially via direct competition, and this is likely to compromise DNA replication fork stability and progression.

## Discussion

### Prelamin A induces DNA replication fork stalling

The expression of prelamin A in U2OS cells induced both mono-ubiquitination of PCNA and increased formation of Pol η foci, two events that are associated with DNA replication fork stalling and which usually occur following DNA damage by UV-irradiation. Importantly, the expression of prelamin A alone was sufficient to cause both responses, implicating prelamin A in the stalling of DNA replication. Further analysis revealed that the sliding-clamp PCNA and translesion synthesis polymerase η colocalised with γH2AX; clear indications that stalled DNA replication forks had collapsed into DSBs and had stimulated the DNA damage response. This is the first time prelamin A has been shown to directly induce DNA damage via perturbing DNA replication and provides a likely model for how prelamin A induces genomic stability.

### Prelamin A induced DNA replication fork stalling is not due to increased DNA photoproducts but is caused by destabilised mature lamina-PCNA interactions

Prelamin A is recognized as altering chromatin organization, often with cells losing heterochromatic DNA. The observed loss of KAP1 upon prelamin A expression in this study supports this notion, and provided a plausible mechanism for how prelamin A could make cells more susceptible to UV induced pyrimidine dimers, as relaxed chromatin is more sensitive to photoproduct formation. However, our data showed no indication that prelamin A caused thymine dimer formation or made cells more sensitive to UV-irradiation.

Other potential causes of stalled DNA replication include unmovable DNA binding proteins,^33^ unusual DNA secondary structures^50^ and even transcription complexes on the same strand,^51^ but it is not obvious how prelamin A could influence the occurrence of these events. It is poorly understood what determines the fate of DNA replication forks at these alternative barriers to progression but evidence suggests that PCNA will still be mono-ubiquitinated at K164 as this occurs independently of pyrimidine dimers, and instead requires ssDNA and forked DNA structures that will be present at all stalled DNA replication forks.^41^ As an intact nuclear lamina is important for DNA replication, and lamin-PCNA interactions are key to replication fork progression, we investigated if prelamin A expression affected binding of PCNA to the mature nuclear lamina. Our results showed that prelamin A was able to bind to PCNA and reduce PCNA interactions with mature lamins. As prelamin A has a different nuclear localization and distribution compared to lamins A/C, this is likely to impact on DNA replication. Despite inducing genomic instability, prelamin A had no effect on proliferation of U2OS cells, probably as these cells lack p16 and therefore will not initiate cell cycle arrest.^52^ However, the formation of DSBs following stalled DNA replication will likely have severe consequences upon cell function of many different cell types that have normal functioning responses to DNA damage. Unrepaired or inappropriately repaired DSBs generated during S-phase can lead to chromosome aberrations that in turn cause cell death or accelerated senescence as demonstrated in vascular smooth muscle cells.^53^ As vascular smooth muscle cell proliferation is essential for vessel repair, loss of these cells would be detrimental to vascular structure and would give rise to cardiovascular complications.

The concept that prelamin A interferes with DNA replication by destabilisation of PCNA bears similarities with experiments that found the addition of truncated lamin proteins caused the induction of intranuclear lamina aggregates that sequestered PCNA away from replication sites and as a result decreased DNA synthesis.^49^ Together, these findings highlight that non-mature lamin A proteins have a significant influence on this essential DNA replication factor. Additionally, progerin was shown to interfere with collapsed DNA replication forks through incorrect recruitment of DNA repair factors,^54^ but there is no evidence that progerin itself causes failure of DNA replication. Indeed, a recent study reported that progerin exhibited a reduced binding affinity toward PCNA than lamin A,^31^ seemingly counteracting our evidence that non-mature lamin A would out-compete the mature lamina for PCNA binding. It should be noted however, that prelamin A is larger than progerin and is more similar in amino acid composition to mature lamin A, thus will retain elements missing in progerin that may allow its interaction with PCNA to persist.

### Concluding remarks

We have provided evidence that prelamin A interferes with DNA replication fork stability and that sequestering of PCNA away from its orthodox interactions with the lamina cause replication stalling and eventual fork collapse that will introduce DSBs into the genome. This occurrence, alongside known defects in the DNA damage response associated with prelamin A accumulation would hinder the ability for cells to maintain homeostasis and eventually accelerate tissue degeneration. In this respect, it may be that prelamin A accumulation that occurs in vascular smooth muscle cells during natural aging^12,16^ also induces genomic instability by obstructing DNA replication and that this could be a contributing factor to cardiovascular aging.

## Supplementary Material

KNCL_A_1239685_Supplementary_Figures.zip

KNCL_1239685_Supplemental_Materials.zip
