# Supplementary figures and images for: Disruption of PCNA-lamins A/C interactions by prelamin A induces DNA replication fork stalling
Source: Nucleus. 2016 Sep 27;7(5):498–511. doi: 10.1080/19491034.2016.1239685 (PMC5120601; doi:10.1080/19491034.2016.1239685)

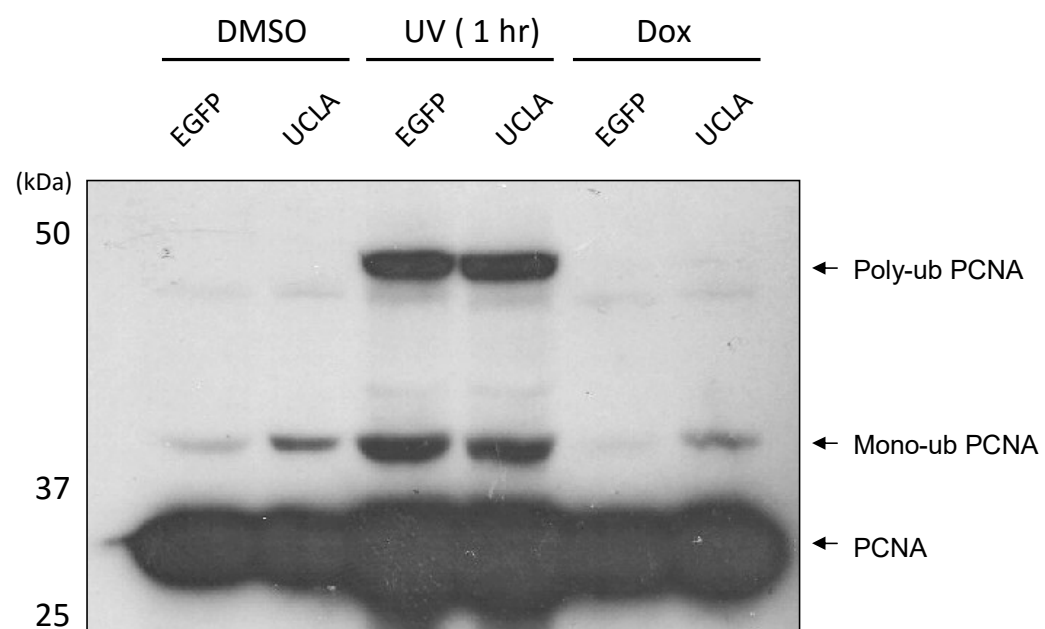

Figure S1

**A**

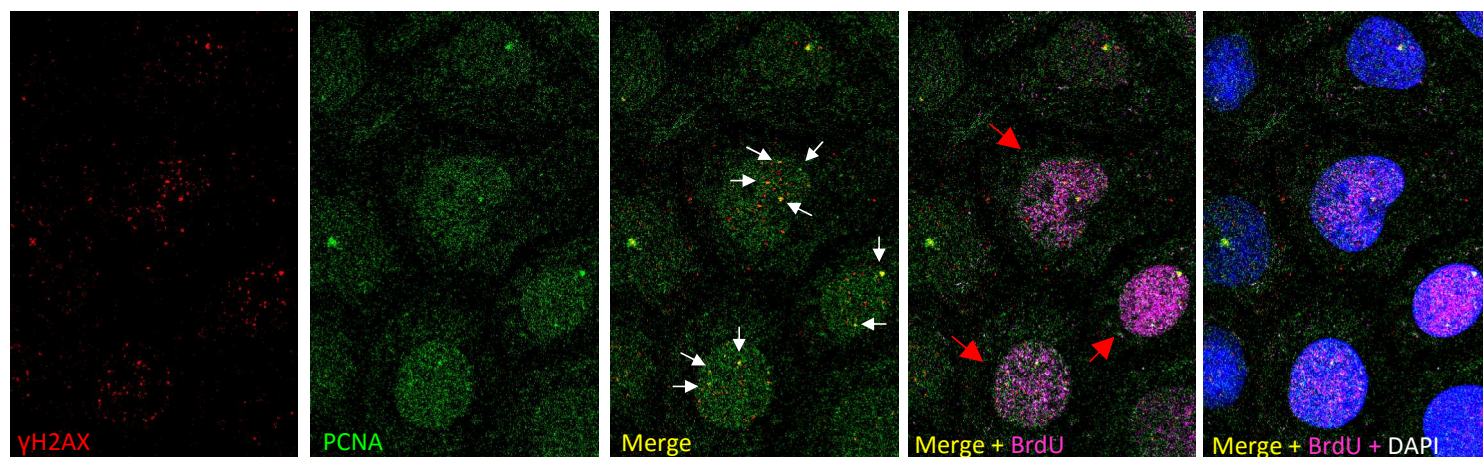

**B**

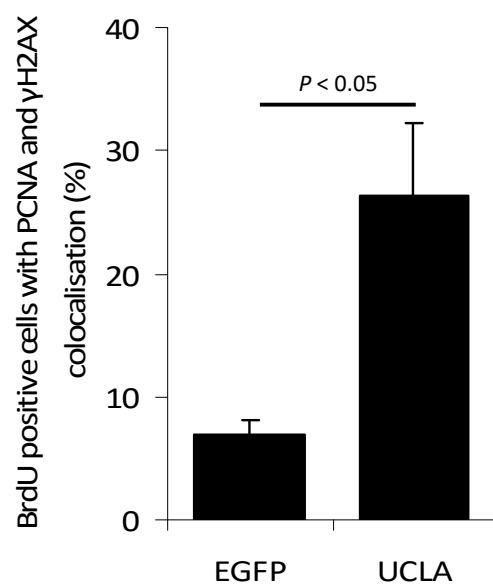

Figure S2

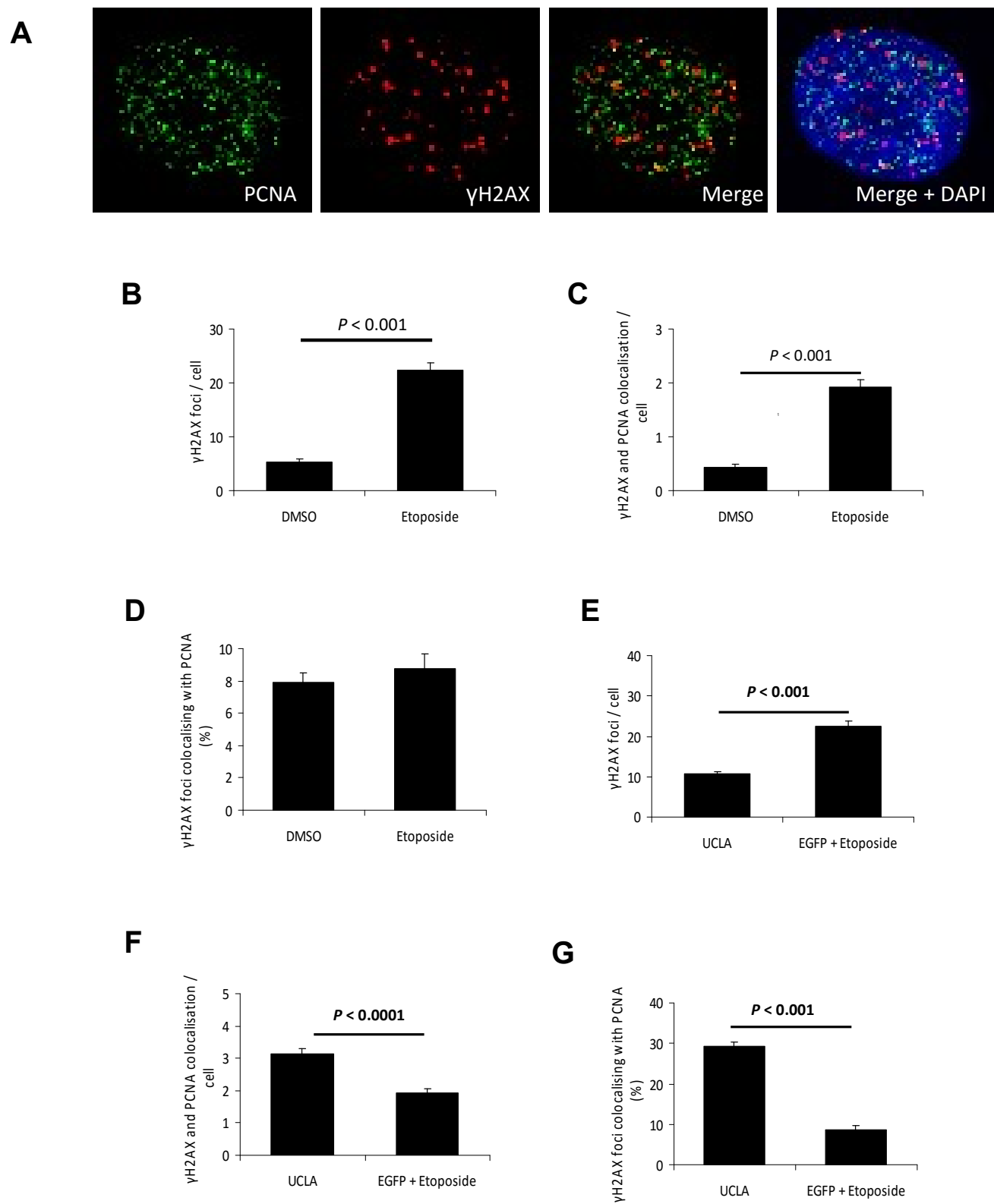

Figure S3

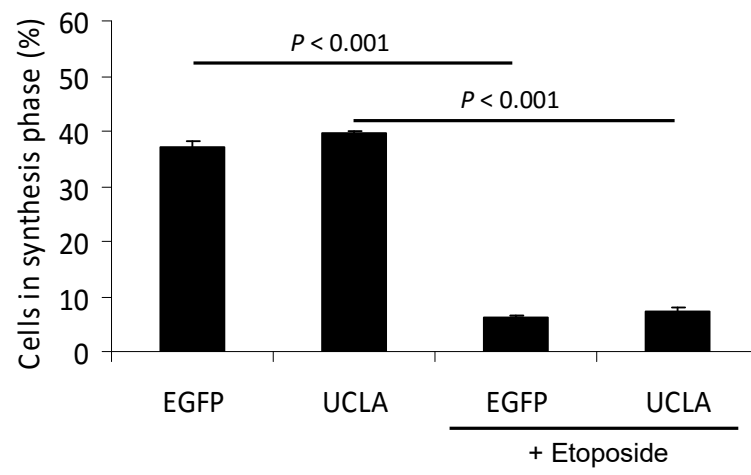

Figure S4

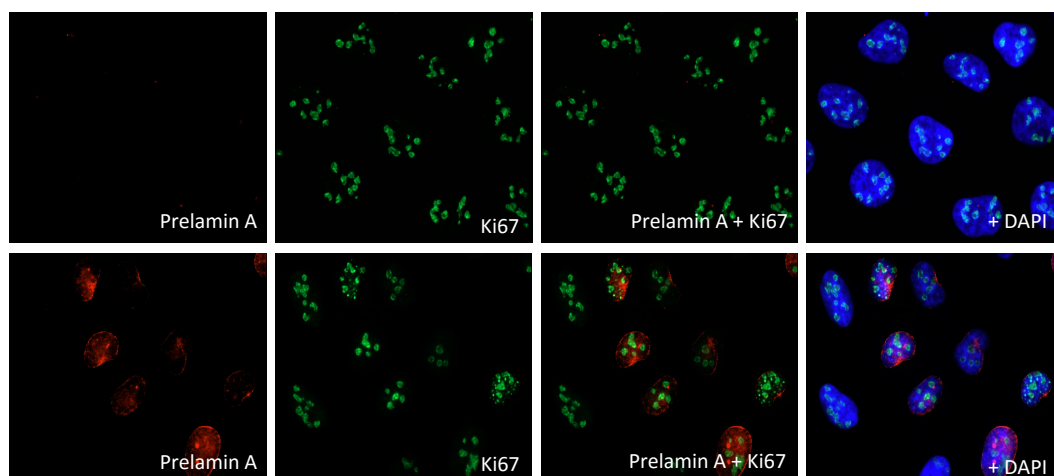

Figure S5

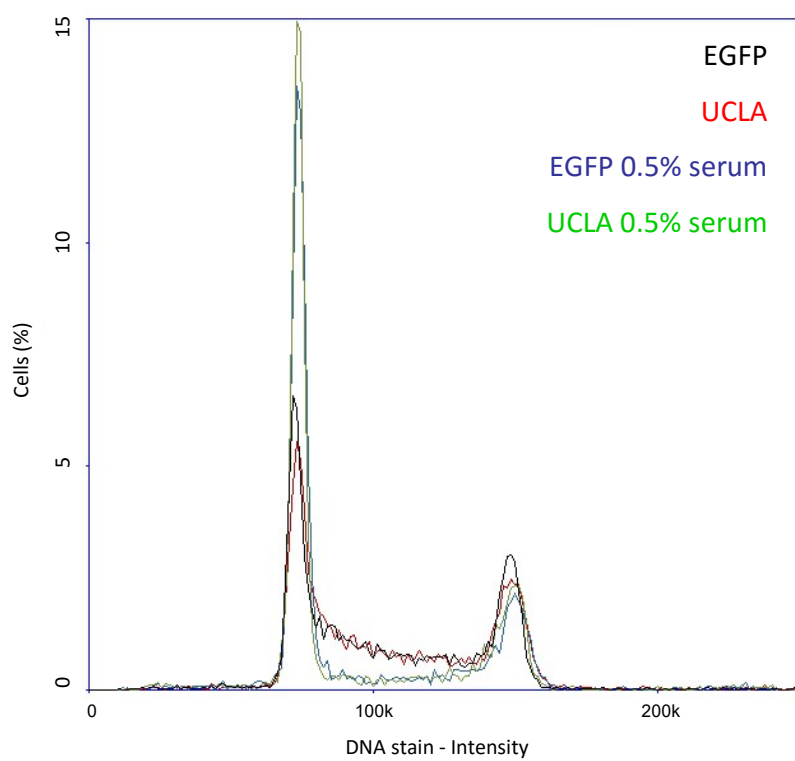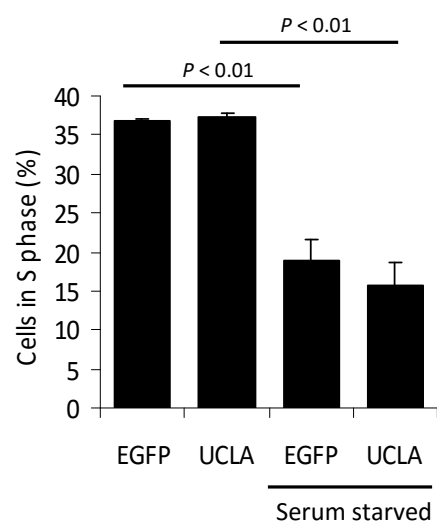

Figure S6

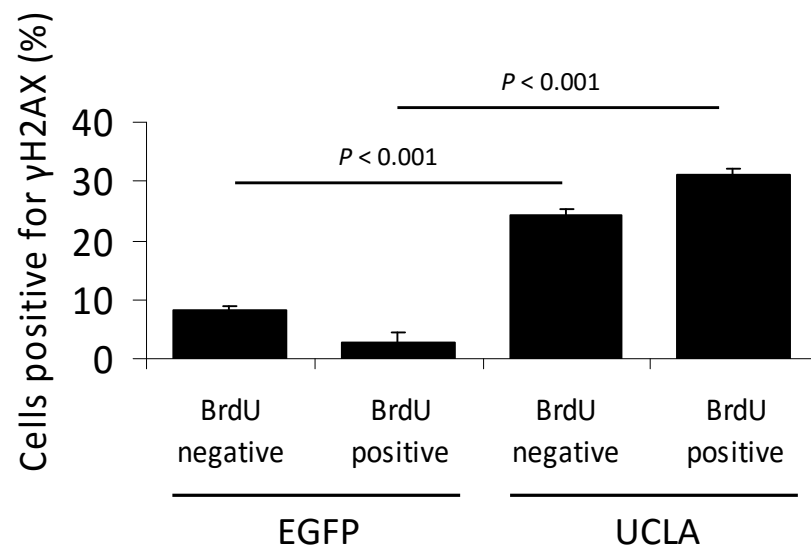

Figure S7

**A**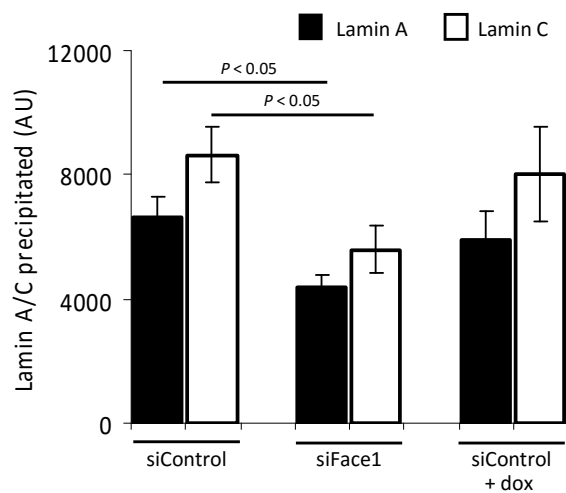**B**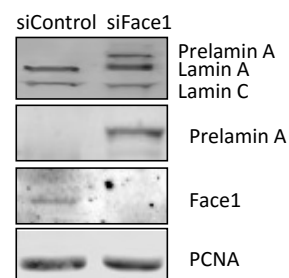**C**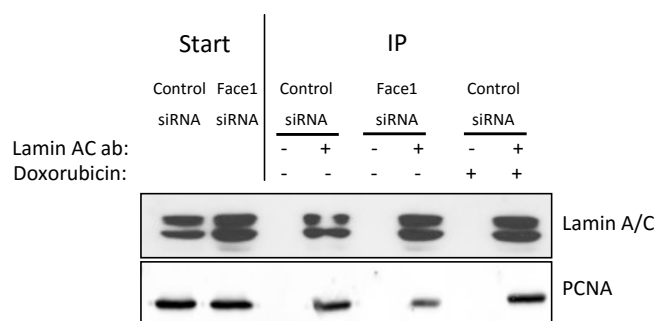**D**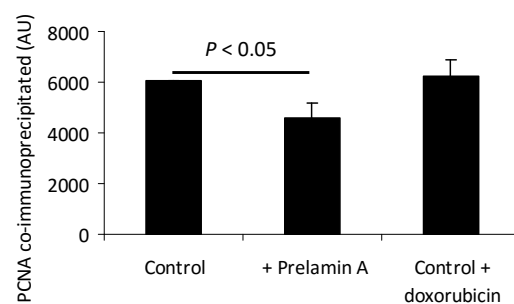

Figure S8

Supplement: KNCL_A_1239685_Supplementary_Figures.zip [file kncl-07-05-1239685-s001.zip › 2016NUCLEUS0049R-f02-z-4c.pdf]
