# Supplementary material for: Disruption of PCNA-lamins A/C interactions by prelamin A induces DNA replication fork stalling
Source: Nucleus. 2016 Sep 27;7(5):498–511. doi: 10.1080/19491034.2016.1239685 (PMC5120601; doi:10.1080/19491034.2016.1239685)
Supplement: KNCL_1239685_Supplemental_Materials.zip [file kncl-07-05-1239685-s002.zip › KNCL_1239685_Supplemental_Materials.doc]

**SUPPLEMENTARY**

S1. **Over-exposed image of a Western blot showing total PCNA and mono-ubiquitinated PCNA in U2OS cells.** The mono-ubiquitinated band was used as part of Figure 1B.

S2. **Triple-staining immunofluorescence showing co-localisation of PCNA and γH2AX in BrdU positive U2OS cells expressing prelamin A.**

**A** U2OS cells were treated with UCLA and then grown in media supplemented with BrdU. Immunofluorescence was then used to visualise PCNA (red), γH2AX (green), and BrdU (purple). DAPI (blue is also shown). Cells that contained the most number of PCNA-γH2AX colocalisations (shown with white arrows) were positive for BrdU (indicated with red arrows), indicating cells in S-phase. Fewer but generally larger foci were seen in non-BrdU cells that may show persistent damage that occurred during S-phase and has not been repaired, therefore is still present after DNA synthesis finished.

**B** Statistical analysis showing the percentage of BrdU positive U2OS cells expressing EGFP or UCLA that have colocalisation of PCNA and γH2AX. *n* > 100 cells from 3 independent experiments, standard errors are shown.

S3. **Etoposide treatment increases amount of γH2AX but does not increase the percentage of γH2AX that is colocalising with PCNA.**

**A** IF showing PCNA (green) and γH2AX (red) in a U2OS cell treated with etoposide. It is evident there is very minimal colocalisation between both foci. DAPI is shown in blue.

**B** Statistical analysis of total γH2AX foci per cell of U2OS cells treated with DMSO or etoposide. *n* > 100 cells from 3 independent experiments, standard errors are shown.

**C** Statistical analysis of incidence of γH2AX and PCNA colocalisation per U2OS cell treated with DMSO or etoposide. The significant difference is likely to occur as there are approximately 4 x the amount of γH2AX in etoposide treated cells, thereby increasing the likelihood of random foci overlap. *n* > 100 cells from 3 independent experiments, standard errors are shown.

**D** Statistical analysis showing percentage of	γH2AX that is colocalising with PCNA in DMSO treated and etoposide treated U2OS cells. Treatment with etoposide did not increase this association. *n* > 100 cells from 3 independent experiments, standard errors are shown.

**E** Statistical analysis comparing total γH2AX foci in UCLA expressing U2OS cells and EGFP expressing U2OS cells that have been treated with etoposide. *n* > 100 cells from 3 independent experiments, standard errors are shown.

**F** Statistical analysis of incidence of γH2AX and PCNA colocalisation per cell of U2OS cells expressing UCLA and cells expressing EGFP that were treated with etoposide. *n* > 100 cells from 3 independent experiments, standard errors are shown.

**G** Statistical analysis of percentage of γH2AX colocalising with PCNA in U2OS cells expressing UCLA and U2OS cells expressing EGFP that were treated with etoposide. Etoposide treatment did not cause the increase in γH2AX associated with PCNA seen in UCLA expressing cells. *n* > 100 cells from 3 independent experiments, standard errors are shown.

S4. **Quantification of FACS data taken from U2OS cells expressing EGFP or UCLA**. The percentage of cells in DNA synthesis phase (S-phase) was measured for cells with and without DNA damage treatment (etoposide). Expression of UCLA did reduce the number of U2OS cells in S phase. Standard errors are shown.

S5. **Immunofluorescence analysis of Ki67 in U2OS cells treated with EGFP or UCLA.** Prelamin A (red) and Ki67 (green) are shown. DAPI is also shown (blue). Expression of UCLA did not affect Ki67 staining, supporting the fact that the accumulation of prelamin A does not reduce cell division of U2OS cells.

S6. **Serum starving cells reduces levels of cells in s-phase.** To confirm that cells grown in 0.5% serum media had decreased levels of cells in s-phase we performed cell-cycle analysis using a Nucleocounter. The plot on the right shows an example of the data and it can be seen expression of UCLA does not affect U2OS cell cycle, however when serum is reduced from 10% to 0.5% there is a significant decrease in cells in s-phase and an increase of cells in G1 (graph on right). Standard errors are shown, *n* = 3.

S7. **Prelamin A causes increased levels of DNA damage in BrdU positive U2OS cells.** Statistical analysis of IF experiment shown in Fig. 4D. U2OS cells were treated with EGFP or UCLA (prelamin A) and then treated with BrdU for 2 hours. DNA damage was determined by staining for γH2AX (cells with 5 or more foci were considered positive for DNA damage). Although prelamin A increased general levels of γH2AX increased in both BrdU negative and BrdU positive cells, the most significant increase was in BrdU positive cells from approximately 5% in controls to 30% in UCLA treated cells. This implicates prelamin A in the induction of DNA damage in cells that are in s-phase.

S8. **Detection of PCNA-lamin A/C interactions.**

**A** Analysis of band intensities from immunoprecipitation experiment shown in Fig. 4G.

**B-D** Reverse immunoprecipitation of experiment shown in Fig. 4G. U2OS cells were treated with control or Face1 siRNA (prelamin A accumulation) and anti-lamin A/C antibody was used to precipitate lamin A/C and interacting proteins. We also analysed if DNA damage affected interactions by treating cells with doxorubicin prior to immunoprecipitation. **B** Start lysates prior to immunoprecipitation experiments showing siFace1 reduces levels of Face1 and as a result increases levels of prelamin A. **C** Immunoprecipitation results showing the presence of prelamin A reduces co-immunoprecipitation of PCNA when lamin A/C is used as bait. **D** Quantification of lamin A/C-PCNA immunoprecipitation assays. Prelamin A was found to significantly reduce the amount of PCNA pulled-down with lamin A/C and this was independent of DNA damage. *n* = 3, standard errors are shown.
